# Thoracic Duct Embolization for Postoperative Lymphatic Fistula

**DOI:** 10.1155/2022/7510793

**Published:** 2022-05-29

**Authors:** Ayhan Erdemir, Murat Dökdök, Kemal Raşa

**Affiliations:** ^1^Department of General Surgery, Anadolu Medical Center Hospital, Turkey; ^2^Department of Radiology, Anadolu Medical Center Hospital, Turkey

## Abstract

Lymphatic fistula as a rare entity particularly emerges after head and neck cancer surgery. It may lead to delayed wound healing, electrolyte loss, malnutrition, dehydration, and immune suppression. Therefore, rapid diagnosis and treatment are of the utmost importance. We aimed to present a lymphatic fistula case who was treated successfully by lymphangiography with percutaneous embolization of the thoracic duct. We believe that minimally invasive techniques could be an alternative to the conservative approach as the first-line choice.

## 1. Introduction

The largest lymphatic vessel in the body is the thoracic duct (TD), and it drains about 75% of the body's lymph fluid [1]. Although it has a highly variable anatomy, it is a superior continuation of cisterna chyli (CC) and ascends two to three cm above the clavicle before turning and descending to the drainage site at the venous junction. During surgical intervention of the left neck, inadvertent injuries may lead to lymphatic fistulas which increase the risk of infection, hypovolemia, electrolyte imbalance, and malnutrition [2].

There is still no gold standard for management and can range from conservative to surgical approaches. A conservative approach is usually preferred, especially for low output fistulas (<500 ml/day), but high output fistulas may necessitate surgical intervention [3]. However, there is no agreement on the timing and operative steps of the surgery.

Besides conservative approaches and surgery, interventional radiological procedures have been increasingly applied in recent years. In this report, we present a case of a lymphatic fistula formed after cervical lymph node excision. As it was refractory to the treatment, the lymphatic fistula was treated with percutaneous embolization through TD.

## 2. Case Presentation

**Figure 1:**
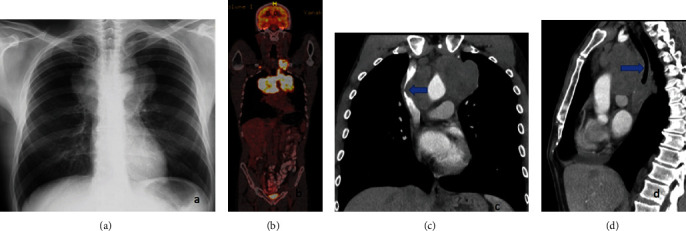
FDG avid lymph nodes in the left supraclavicular region extending to the anterior mediastinum.

A 50-year-old, heavy-smoker male patient presented with weakness, fatigue, cough, night sweating, fever, and dyspnea and had lost two kg during the last two months. Besides his cachectic appearance, physical examination revealed multiple pathologic cervical lymphadenopathies. PET-CT reported FDG avid lymph nodes with the biggest measuring 68 × 55 × 29 mm (SuvMax: 19) and 85 × 150 × 95 mm (SuvMax: 28) in the left supraclavicular region extending to the anterior mediastinum (Figure 1). The laboratory investigations were otherwise normal. An excisional biopsy was recommended by a medical oncologist with the differential diagnoses of mediastinal seminoma, thymic carcinoma, and lymphoma.

**Figure 2:**
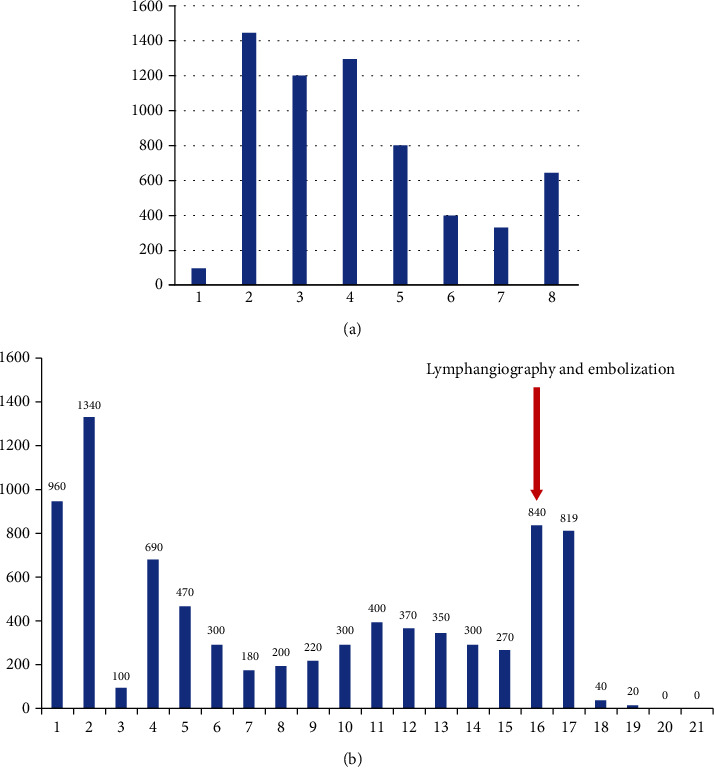
(a) Lymphatic fistula output in the early postoperative period. (b) Lymphatic fistula output after first lymphangiography.

**Figure 3:**
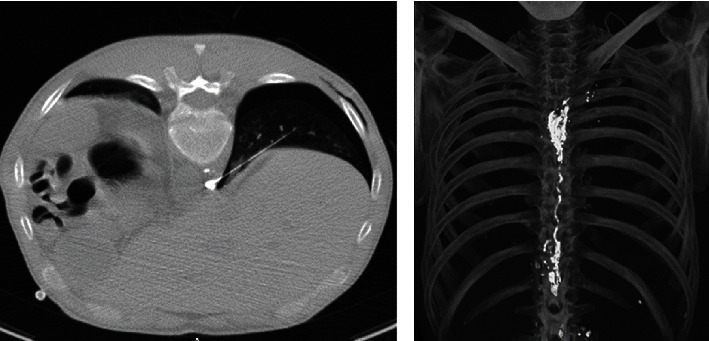
CT confirmation of the fistula site embolization and closure.

On the postoperative second day, a lymphatic fistula was observed with an output up to 1400 ml daily (Figure 2(a)). Considering the morbidity and mortality risk of reexploration, we preferred a conservative approach in the first place. At the end of the first week, the patient was consulted with interventional radiology. After intranodal lymphangiography of the groin with 10 ml lipiodol using a 25-gauge spinal needle, the lymphatic fistula was confirmed at the neck. However, only a few droplets due to the compression of the mediastinal mass were seen in the late images reaching the fistula site. Besides, multiple channels of lymphatic vessels, without forming CC, continuing as TD distally was observed. We decided to continue conservative treatment with a pressure bandage, TPN, and Sandostatin 3 × 100 mc sc. Although documented daily output was fairly decreased, the fistula persisted (Figure 2(b)). Sixteen days after the first lymphangiography, transthoracic translobar puncture of TD directly using 21-gauge 20 cm Chiba needle (Cook Inc. Bloomington, IN) under computed tomography (CT) fluoroscopy guidance was achieved. After visualization of the TD and collateral lymphatics with 1 ml Lipiodol injection, embolization was performed with 4 ml of 1 : 1 N-Butyl cyanoacrylate (n-BCA) (Truefill Cordis, Johnson & Johnson, Warren NJ) and Lipiodol mixture. The fistula site and closure were then confirmed with CT (Figure 3). The fistula closed within two days after the intervention and in due time etoposide and cisplatin-based chemotherapy was initiated for thymic carcinoma diagnosis.

## 3. Discussion

TD injuries and lymphatic fistulas are rare surgical complications, which may arise mostly after lateral cervical dissection for malignant or metastatic diseases and in patients with a previous radiotherapy history [4]. The neck region, especially the lower jugular area, should be carefully examined intraoperatively, and the detected lymphatic leaks should be ligated and controlled. Trendelenburg position and the Valsalva maneuver during anesthesia may facilitate visualization of the leak [5].

Due to protein, fat, essential elements, and lymphocyte loss, hypovolemia, electrolyte imbalance, malnutrition, and immunosuppression may evolve. These adverse effects are more pronounced in high-output fistulas. Expanding lymphatic fluid in the surgical site has also a negative impact on skin flaps and impairing their perfusion pressure effect may even cause flap necrosis. This fluid can also drain into the mediastinum and pleura, and related complications may develop. Since lymphatic fluid drainage increases with physical activity, conservative treatment should be started with activity restriction. High-volume fluid, protein, and electrolyte losses should be closely monitored and replaced [4]. Protein-rich and medium-chain fatty acid-containing diets should also be preferred. Medium-chain fatty acid-containing foods, total parenteral nutrition, drainage, repetitive aspiration, negative pressure wound care, and octreotide therapy are the standards for a conservative approach.

Although surgery is recommended in a high-output fistula (>500 ml/day), it remains subjective per the clinical conditions of the patient and with the surgeon's experience [2]. Considering the comorbidities and respiratory distress due to the mediastinal mass, we concluded that the risk of re-do TD ligation surgery would be substantially high.

Intranodal lipiodol lymphangiography has been described recently for the diagnosis and treatment of lymphatic injury in the abdomen and thorax. In persistent cases, thoracic duct catheterization via a cisterna chyli and embolization with coils or liquid agents could be performed [6–9]. We performed embolization with a 1 : 1 mixture of n-BCA-lipiodol, following direct transthoracic translobar thoracic duct puncture because of the plexiform variant of CC. To our knowledge, this technique has not been described previously in the literature.

Herein, we present a lymphatic fistula case that was treated successfully by lymphangiography with percutaneous embolization of the thoracic duct. We concluded that minimally invasive techniques could be used as a therapeutic alternative to redo duct ligation surgery and even might be considered as an alternative to the conservative approach as the first-line choice.

## Data Availability

The data used to support the findings of this study are included within the article.

## References

[1] Phang K. L., Bowman M., Phillips A., Windsor J. (2014). Review of thoracic duct anatomical variations and clinical implications. *Clinical Anatomy*.

[2] Parmeggiani D., Gualtieri G., Terracciano G. (2021). Prolonged iatrogenic thoracic duct chylous fistula in neck surgery: conservative management or surgery? A literature review. *Scandinavian Journal of Surgery*.

[3] Lee Y. S., Nam K. H., Chung W. Y., Chang H. S., Park C. S. (2010). Postoperative complications of thyroid cancer in a single center experience. *Journal of Korean Medical Science*.

[4] Crumley R. L., Smith J. D. (1976). Postoperative chylous fistula prevention and management. *The Laryngoscope*.

[5] Cernea C. R., Hojaij F. C., De Carlucci D. (2012). Abdominal compression: a new intraoperative maneuver to detect chyle fistulas during left neck dissections that include level IV. *Head & Neck*.

[6] van Goor A. T., Kröger R., Klomp H. M., de Jong M. A. A., van den Brekel M. W. M., Balm A. J. M. (2007). Introduction of lymphangiography and percutaneous embolization of the thoracic duct in a stepwise approach to the management of chylous fistulas. *Head & Neck*.

[7] Patel N., Lewandowski R. J., Bove M., Nemcek A. A., Salem R. (2008). Thoracic duct embolization: a new treatment for massive leak after neck dissection. *The Laryngoscope*.

[8] Chen C. Y., Chen Y. H., Shiau E. L., Liang H. L., Chang H. S., Chen H. C. (2016). Therapeutic role of ultrasound-guided intranodal lymphangiography in refractory cervical chylous leakage after neck dissection: report of a case and review of the literature. *Head & Neck*.

[9] Flores-Funes D., Miguel Perelló J. A., Capel-Alemán A., Flores-Pastor B. M. (2021). Embolizacion percutanea del conducto toracico como alternativa terapeutica de la fistula quilosa tras cirugia tiroidea. *Endocrinología, Diabetes y Nutrición (English ed.)*.

